# Potential Change in Insulin Out-of-Pocket Spending Under Cost-Sharing Caps Among Pediatric Patients With Type 1 Diabetes

**DOI:** 10.1001/jamapediatrics.2020.1065

**Published:** 2020-07-27

**Authors:** Kao-Ping Chua, Joyce M. Lee, Rena M. Conti

**Affiliations:** 1 Susan B. Meister Child Health Evaluation and Research Center, Department of Pediatrics, University of Michigan Medical School, Ann Arbor; 2 Division of Pediatric Endocrinology, Department of Pediatrics, University of Michigan Medical School, Ann Arbor; 3 Institute for Health System Innovation and Policy, Questrom School of Business, Department of Markets, Public Policy, and Law, Boston University, Boston, Massachusetts

## Abstract

This cross-sectional study estimates the potential change in insulin out-of-pocket spending among privately insured children and young adults with type 1 diabetes if national caps were implemented.

To improve insulin affordability, several states and insurers have implemented cost-sharing caps. For example, Colorado implemented a $100 cap for a 30-day insulin supply, while the insurer Cigna implemented a $25 cap.^1,2,3^ We estimated the potential change in insulin out-of-pocket spending among privately insured children and young adults with type 1 diabetes if national $25 and $100 caps were implemented.

## Methods

We conducted a cross-sectional analysis of the 2018 IBM MarketScan Commercial Database, which includes nonelderly patients across the US with private insurance coverage from medium to large employers.^4^ We included patients aged 1 to 21 years with continuous enrollment in 2018, 1 or more type 1 diabetes diagnosis codes in 2017 (to limit the population to established patients), and 1 or more insulin pharmacy claims in 2018. The Institutional Review Board of the University of Michigan Medical School exempted this study from review, and informed consent was not required. This article followed the Strengthening the Reporting of Observational Studies in Epidemiology (STROBE) reporting guideline.

We calculated mean and median annual insulin out-of-pocket spending (sum of copays, deductibles, and coinsurance) without caps. We assessed changes under national caps that constrained out-of-pocket spending for a 30-day insulin supply to $25 or $100, assuming caps were applied per prescription. Under a $25 cap, we constrained out-of-pocket spending for insulin prescriptions to $25 if days supplied was 30 days or less, $50 if days supplied was between 31 and 60 days, $75 if days supplied was between 61 and 90 days, and so on. Under a $100 cap, the corresponding maximums were $100, $200, and $300, respectively. Under each cap, we determined the proportion of patients who would benefit and changes in annual out-of-pocket spending among those who would benefit. We repeated analyses among high-deductible health plan (HDHP) enrollees and nonenrollees.

We compared proportions and changes in out-of-pocket spending using χ^2^ and Wilcoxon rank sum tests. Two-sided *P* values less than .05 were considered statistically significant. Analyses were conducted using SAS version 9.4 (SAS Institute).

## Results

Of 13 255 patients with continuous enrollment in 2018 and at least 1 type 1 diabetes diagnosis code in 2017, 12 185 (91.9%) had 1 or more insulin claims in 2018. Of these patients, 2088 (17.1%) were aged 1 to 11 years, 5054 (41.5%) were aged 12 to 17 years, and 5043 (41.4%) were aged 18 to 21 years, and 3116 (25.6%) were HDHP enrollees. Patients had a mean (SD) of 7.7 (4.8) annual insulin claims. The mean (SD) days supplied per claim was 49.3 (28.4) days.

As shown in the Table, the mean (SD) annual out-of-pocket spending was $494 (640). The median (25th and 75th percentile) spending was $308 (120-638). Annual out-of-pocket spending exceeded $1000 for 1538 patients (12.6%) and comprised copays ($303 [61.3%]), deductibles ($77 [15.6%]), and coinsurance ($114 [23.1%]). A $25 cap would benefit 7302 patients (59.9%); their annual out-of-pocket spending would decrease from $741 to $261 (mean decrease, $481). A $100 cap would benefit 2151 patients (17.7%); their annual out-of-pocket spending would decrease from $1343 to $786 (mean decrease, $558) (Figure).

**Table.** Annual Insulin Out-of-Pocket (OOP) Spending Under $25 and $100 Monthly Caps Among Pediatric Patients With Type 1 Diabetes Overall and by High-Deductible Health Plan (HDHP) Enrollment^a^^,^^b^

Category	Outcome	Cap, Mean (SD), $	
		$25	$100
**All patients (N = 12 185)**^c^			
Patients who would benefit from cap	No. (%)	7302 (59.9)	2151 (17.7)
	Annual OOP spending without cap	741 (720)	1343 (1017)
	Annual OOP spending with cap^d^	261 (139)	786 (422)
	Decrease in annual OOP spending with cap^e^	481 (683)	558 (873)
Patients who would not benefit from cap	No. (%)	4883 (40.1)	10 034 (82.3)
	Annual OOP spending with or without cap	124 (134)	312 (296)
All patients	Annual OOP spending without cap	494 (640)	494 (640)
	Annual OOP spending with cap^d^	206 (153)	395 (369)
	Decrease in annual OOP spending with cap^e^	288 (579)	98 (424)
**HDHP enrollees (n = 3116)**			
HDHP enrollees who would benefit from cap	No. (%)	2277 (73.1)	994 (31.9)
	Annual OOP spending without cap	866 (752)	1342 (879)
	Annual OOP spending with cap^d^	237 (133)	793 (411)
	Decrease in annual OOP spending with cap^e^	628 (706)	549 (735)
HDHP enrollees who would not benefit from cap	No. (%)	839 (26.9)	2122 (68.1)
	Annual OOP spending with or without cap	39 (74)	316 (330)
All HDHP enrollees	Annual OOP spending without cap	643 (741)	643 (741)
	Annual OOP spending with cap^d^	184 (149)	468 (421)
	Decrease in annual OOP spending with cap^e^	459 (664)	175 (488)
**Non-HDHP enrollees (n = 9069)**			
Non-HDHP enrollees who would benefit from cap	No. (%)	5025 (55.4)	1157 (12.8)
	Annual OOP spending without cap	685 (697)	1344 (1122)
	Annual OOP spending with cap^d^	271 (141)	779 (431)
	Decrease in annual OOP spending with cap^e^	414 (662)	565 (976)
Non-HDHP enrollees who would not benefit from cap	No. (%)	4044 (44.6)	7912 (87.2)
	Annual OOP spending with or without cap	141 (137)	311 (287)
All non-HDHP enrollees	Annual OOP spending without cap	443 (592)	443 (592)
	Annual OOP spending with cap^d^	213 (153)	371 (346)
	Decrease in annual OOP spending with cap^e^	229 (534)	72 (396)

^a^ Insulin products included insulin aspart, degludec, detemir, glargine, glulisine, lispro, NPH, inhaled human insulin, and regular human insulin.

^b^ HDHP enrollees were defined as patients with MarketScan variable PLANTYP 8 or 9 (corresponding to consumer-driven health plan and qualified HDHP) throughout 2018.

^c^ Patients were required to have continuous enrollment in 2018, 1 or more insulin claims in 2018, and 1 or more claims in 2017 containing an *International Statistical Classification of Diseases, Tenth Revision, Clinical Modification* diagnosis code for type 1 diabetes (E10). Continuous enrollment in 2017 was not required. We required that patients have a type 1 diabetes diagnosis code in 2017 to exclude patients who may have lower annual OOP spending for insulin because they were newly diagnosed later during 2018. In sensitivity analyses, results were virtually identical when requiring a type 1 diabetes diagnosis code only in 2018 or in both 2017 and 2018.

^d^ We made several assumptions. First, we assumed caps were applied per prescription, meaning that patients filling prescriptions for 2 different types of insulin would be subjected to 2 separate caps. This approach reflects how many states have implemented caps, including Colorado. Second, we assumed the number of insulin claims per patient remained constant, as prior literature suggests that insulin demand is inelastic. Third, we assumed that capping insulin out-of-pocket spending would not affect whether and when patients met deductibles or annual out-of-pocket maximums. Finally, we assumed that self-insured employers—who are typically exempted from state-imposed caps under the Employee Retirement Income Security Act—would not be exempted because the cap we modeled was national (ie, instituted by federal legislation).

^e^ May not equal difference between the numbers in the 2 rows above due to rounding error.

**Figure.**
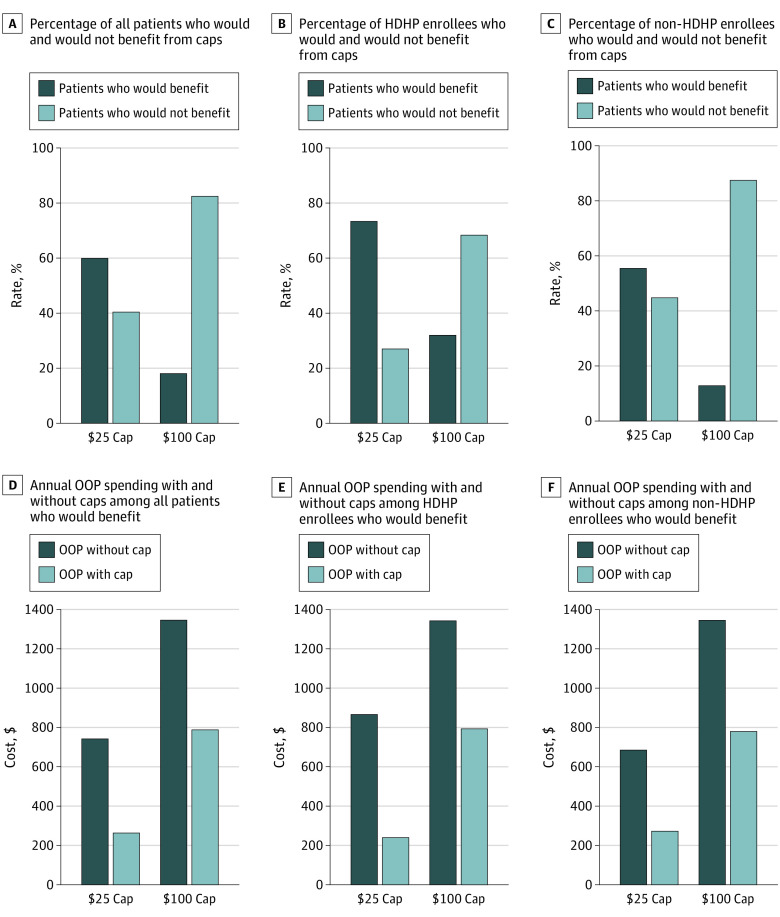
Potential Change in Insulin Out-of-Pocket (OOP) Spending Under Cost-Sharing Caps Among Pediatric Patients With Type 1 Diabetes Overall and by High-Deductible Health Plan (HDHP) Enrollment Data derived from the 2018 IBM MarketScan Commercial Database.^4^

Among 3116 HDHP enrollees, the mean (SD) annual out-of-pocket spending was $643 (741). The median (25th and 75th percentile) spending was $428 (102-917). Annual out-of-pocket spending exceeded $1000 for 677 patients (21.7%) and comprised copays ($238 [37.0%]), deductibles ($171 [26.5%]), and coinsurance ($235 [36.5%]). A $25 cap would benefit a greater proportion of HDHP enrollees than nonenrollees (2277 of 3116 [73.1%] vs 5025 of 9069 [55.4%]; *P* < .001). Among those who would benefit, annual out-of-pocket spending would decrease more among HDHP enrollees ($628 vs $414; *P* = .02). A $100 cap would benefit a greater proportion of HDHP enrollees than nonenrollees (994 of 3116 [31.9%] vs 1157 of 9069 [12.8%]; *P* < .001). Among those would benefit, annual out-of-pocket spending would decrease to similar degrees ($549 vs $565; *P* = .60) (Figure).

## Discussion

In 2018, mean out-of-pocket spending for insulin among privately insured children and young adults with type 1 diabetes was $494; for 1 in 8, spending was more than $1000. For perspective, 40% of those in the US lacked the savings to pay for a $400 emergency in 2018.^5^ For 60% and 18% of patients, out-of-pocket spending would decrease under national $25 and $100 caps, respectively. Caps would benefit HDHP enrollees more than nonenrollees.

Caps have limitations. They do not address rising insulin prices, improve insulin affordability for the uninsured, or limit cost-sharing for diabetes-related supplies, such as insulin pumps. Additional policies are needed to alleviate the financial burden among patients with type 1 diabetes.
